# Who Invited the Celebrities? Psychiatric Experience in Charles Bonnet Syndrome: A Case Report

**DOI:** 10.1192/j.eurpsy.2026.11572

**Published:** 2026-07-31

**Authors:** M. Calvo Valcárcel, M. A. Andreo Vidal, C. Rodríguez Valbuena, A. Monllor Lazarraga, B. Rodriguez Rodriguez, M. Queipo de Llano de La Viuda

**Affiliations:** Psychiatry, Hospital Clínico Universitario de Valladolid, Valladolid, Spain

## Abstract

**Introduction:**

Charles Bonnet Syndrome (CBS) is characterized by the presence of complex visual hallucinations, such as objects or people, triggered by vision deprivation in the absence of cerebral, psychiatric, pharmacological, or systemic disorders. It is believed to result from brain escape phenomena due to cerebral deafferentation. Most affected patients are elderly individuals, although cases have also been described in various age groups. It occurs in 15% of individuals with visual loss, with a predominance in females around 80 years of age.

**Objectives:**

This case aims to analyze the clinical presentation in a patient with a suspected diagnosis of Charles Bonnet Syndrome.

**Methods:**

A thorough review of the literature on CBS and the psychiatric symptoms that could complicate its diagnosis has been carried out to support the presentation of the case.

**Results:**

We present the case of an 84-year-old woman with a long history of psychiatric illness due to persistent depressive disorder was recently diagnosed with mild cognitive impairment. She has no ophthalmological history beyond age-related changes. She presented to the Emergency Department with visual hallucinations in the form of “famous television presenters,” which she partially criticizes while being mostly aware of their unreality. The hallucinations are not accompanied by significant somatic anxiety or affective impact. Delirium, substance intoxication, or medications causing the symptoms have been ruled out. She is currently under follow-up to exclude other causes. No pharmacological treatment has been administered.

**Conclusions:**

The diagnosis of CBS is made by exclusion and is particularly challenging, as hallucinatory episodes can vary in content, duration, and frequency, even within the same patient, necessitating a multidisciplinary approach involving neurologists, psychiatrists, and ophthalmologists to avoid misdiagnosis. Differential diagnosis should include conditions such as delirium, Lewy body dementia, toxic substance intoxication, Parkinson’s disease, endocrine-metabolic disorders, and migrainous aura, among others. It is important to involve the family in managing the syndrome to reinforce the recognition of the unreality of the hallucinations in patients. Antipsychotic treatment may be effective only if the condition causes significant distress.

**Disclosure of Interest:**

None Declared

